# Immune-Mediated Hemolytic Anemia in Cats with Feline Infectious Peritonitis

**DOI:** 10.3390/pathogens14070660

**Published:** 2025-07-04

**Authors:** Petra Černá, Marieke Knies, Marleen Assink, Samantha Evans, Séverine Tasker, Danièlle A. Gunn-Moore, Katrin Hartmann, Katharina Buchta, Samantha Taylor, Solène Meunier, Regina Hofmann-Lehmann, Nicole Jacque, Allison Koonce, Casandra Jacobs, Ashley Gillett, Michael R. Lappin

**Affiliations:** 1 Department of Clinical Sciences, Colorado State University, Fort Collins, CO 80523, USA; 2 Department of Clinical Sciences, Faculty of Veterinary Medicine, Utrecht University, 3584 CT Utrecht, The Netherlands; 3 AniCura Specialist Referral Centre Haaglanden, Frijdastraat 20a, 2288 EZ Rijswijk, The Netherlands; 4 IVC Evidensia Small Animal Referral Hospital Arnhem, Meander 10, 6825 MB Arnhem, The Netherlands; 5 Bristol Veterinary School, University of Bristol, Bristol BS40 5DU, UK; s.tasker@bristol.ac.uk; 6 Mars Veterinary Health, Shirley, Solihull B90 4BN, UK; 7 The Royal (Dick) School of Veterinary Studies, University of Edinburgh, Easter Bush Campus, Midlothian EH25 9RG, UK; 8 LMU Small Animal Clinic, Centre for Clinical Veterinary Medicine, LMU Munich, 80539 Munich, Germanyk.buchta@medizinische-kleintierklinik.de (K.B.); 9 International Cat Care Veterinary Society, Tisbury SP3 6HJ, UK; 10 Lumbry Park Veterinary Specialists, Selborne Road, Alton GU34 3H, UK; 11 Clinic for Small Animal Internal Medicine, Vetsuisse Faculty, University of Zurich, Winterthurerstrasse 260, CH-8057 Zurich, Switzerland; solene.meunier@uzh.ch; 12 Clinical Laboratory, Department of Clinical Diagnostics and Services, Center for Clinical Studies, Vetsuisse Faculty, University of Zurich, CH-8057 Zurich, Switzerland; 13 Independent Researcher, San Jose, CA 95113, USA; 14 Pleasant Valley Veterinary Clinic, Little Rock, AR 72212, USA; koonce.alli@gmail.com; 15 Desert Veterinary Medical Specialists, Peoria, AZ 85382, USA; 16 The Humane Society of Sarasota County, Sarasota, FL 34237, USA

**Keywords:** FIP, PIMA, IMHA, coronavirus

## Abstract

Feline infectious peritonitis (FIP) is caused by mutated feline coronaviruses. Immune-mediated hemolytic anemia (IMHA) arises due to immune-mediated erythrocyte destruction and can be non-associative or associative with diseases such as FIP. Records of cats with FIP were reviewed to find those with associative IMHA based on exclusion of other causes of anemia and a positive saline agglutination test and/or Coombs test. The inclusion criteria were met for 45 cats (26 (58%) cats with effusive and 19 (42%) with non-effusive FIP). Median hematocrit was 18% (interquartile range [IQR] 13–20). Anemia was non-regenerative in 36 (80%) cats and regenerative in 5 (11%) cats; 4 (9%) cats had no reticulocyte count available. Concurrent thrombocytopenia was present in 18 (40%) cats. All 45 cats were treated with nucleoside analogs, and 44 (98%) cats with glucocorticoids; in 5 (11%) cats, glucocorticoids were added after starting antiviral treatment due to persistent anemia. Median follow-up was 72 days (IQR 14–246); at the time of last follow-up 33 (73%) cats had survived while 12 (27%) had died or were euthanized. Of the 33 surviving cats, 17 achieved remission of both FIP and IMHA. In three cats, FIP remission was achieved, but IMHA relapsed; in one of these, IMHA relapsed twice. FIP relapsed without IMHA in two cats, and both FIP and IMHA relapsed in one cat. In 9 cats the antiviral and glucocorticoid treatment is still ongoing at the time of the publication. Although FIP is likely an uncommon cause of associative IMHA, as more cats with FIP are treated with antiviral therapy, it is important to consider IMHA as a possible cause of anemia in cats with FIP.

## 1. Introduction

Feline infectious peritonitis (FIP) is a disease caused by feline coronavirus (FCoV), although the pathogenesis is still not fully understood [1]. Recent studies have reported the use of antiviral drugs like the nucleoside analogs GS-441524 and molnupiravir to successfully treat FIP [2,3,4,5,6,7,8]. Immune-mediated hemolytic anemia (IMHA) occurs when the body mounts an immune response against antigens expressed on the surface of erythrocytes [9]. In animals with IMHA, the production of antibodies results in the destruction of normal erythrocytes by inappropriate activation of the complement cascade, antibody-dependent cytotoxicity, and/or facilitated phagocytosis in the liver and spleen, resulting in severe anemia. In cats, IMHA can either be non-associative (primary) or be associative (secondary) to an underlying infectious, inflammatory, or neoplastic process [10,11]. Several infections have been documented in cats to cause associative IMHA, including feline leukemia virus (FeLV), feline immunodeficiency virus (FIV), *Leishmania infantum*, *Mycoplasma gatae*, *Mycoplasma haemofelis*, *Babesia felis*, and FIP [10,11,12,13,14,15,16,17]. One study also reported one FIP and IMHA cat to have co-infection with FeLV [14]. While most cats with FIP were euthanized in the past, more cats are now being treated with antiviral drugs. As a result of this, new disease processes that could be associated with FIP are being recognized, for example, IMHA, myocarditis or other cardiac changes, and other comorbidities [18,19]. A case report described a 9-month-old cat diagnosed with FIP that developed left-sided congestive heart failure secondary to hypertrophic cardiomyopathy (HCM) phenotype 4 days after initial diagnosis [19]. Humans with SARS-CoV-2 infections present with a spectrum of clinical presentations and complications, with associative IMHA being increasingly recognized in patients, either while infected or shortly after infection [20]. It has been proposed that SARS-CoV-2 infection could lead to hemolytic anemia directly through cytopathic injury or indirectly through induction of autoantibodies [21]. The objective of this study was to retrospectively report cases of cats with FIP that presented with concurrent IMHA, including the clinical signs, response to treatment, and prognosis.

## 2. Materials and Methods

### 2.1. Case Inclusion

To be included in this study, the FIP diagnosis had to be either (1) confirmed based on positive immunohistochemistry (IHC) for FCoV antigen or positive reverse transcription polymerase chain reaction (RT-PCR) for FCoV RNA, as previously reported [22], or (2) presumed based on compatible history, signalment, clinical signs, bloodwork abnormalities, and positive response to antiviral medications, as described by the Advisory Board for Cat Diseases (ABCD) [23]. The IMHA diagnosis was made according to the ACVIM consensus statement on the diagnosis of IMHA in dogs and cats [24] by a positive saline agglutination test (persistent microscopic or macroscopic autoagglutination after washing) or, if the saline agglutination test was negative, by a positive direct antiglobulin test (DAT, also known as Coombs test). Standard diagnostic tests for other infectious causes of anemia were performed, including FeLV antigen testing, FIV antibody testing, and polymerase chain reaction [PCR]) for hemotropic *Mycoplasma* spp. (hemoplasma)*.*

The participating clinics were contacted and asked to review medical records for cats meeting the entry criteria. The following data were recorded for each case: signalment; clinical examination findings; form/type of FIP; results of CBC and serum biochemical profile; and whether the cat was positive by DAT or agglutination in saline or both, and point-of-care FeLV antigen and FIV antibody testing. Hemoplasma species testing, any cytologic or histologic examinations; findings from thoracic and abdominal imaging; blood type and records of blood product transfusion, and the immunosuppressive drugs or other treatments (e.g., antimicrobials) administered were recorded, if available. Follow-up information was provided for the cats, which included survival (at least 2 weeks after starting antiviral therapy) and response to antiviral and/or glucocorticoid therapy. Relapse was defined for FIP as return of clinical signs or clinicopathological abnormalities when stopping antiviral therapy. Relapse of IMHA was defined as return of clinical signs or clinicopathological abnormalities when stopping or tapering glucocorticoid therapy.

### 2.2. Statistical Analysis

All analyses for descriptive statistical analysis were conducted using statistical software (Excel, Microsoft, Redmond, WA, USA).

## 3. Results

### 3.1. Signalment

A total of 45 cats with FIP met the inclusion criteria for this study, from veterinary hospitals in the following countries: Unites States, United Kingdom, The Netherlands, Germany, and Switzerland. The median age was 1.2 years (interquartile range [IQR] 0.5–2.9). There were 34 (76%) males (22 neutered and 12 intact) and 11 (24%) females (7 spayed females and 4 intact). Most cats were domestic shorthair (23; 51%) or longhair (1), and there were 21 (47%) pedigree cats (8 British Shorthairs, 5 Maine Coons, 2 Devon Rexes, 2 Siberian, 1 Ragdoll, 1 Sacred Birman, 1 Turkish Angora, and 1 Ragdoll/Siamese cross).

### 3.2. Clinical Findings

FIP was classified as effusive in 26 (58%) cats and non-effusive in 19 (42%) cats. Among the effusive cases, 5 had neurologic involvement, and 4 had ophthalmic involvement. Among the non-effusive cases, 3 had neurologic involvement, 4 had ophthalmic involvement, and 3 cats had both. The most common clinical signs were lethargy (34 cats; 76%), hyporexia (29 cats; 64%), enlarged abdomen (11 cats; 24%), weight loss (9; 20%), vomiting (3; 7%), diarrhea (3; 7%), and pica (2; 4%). Reported ocular signs were uveitis (9; 20% cats) and anisocoria (2; 4%). Among neurological signs, the most common sign was ataxia (4 cats; 9%), then seizures (2; 4%), paresis (2; 4%), tremors (2; 4%), hind limb weakness (2; 4%), and generalized weakness (1; 2%). Other clinical signs included lack of appropriate weight gain (1 cat; 2%), behavioral changes (1; 2%), polyuria in (1; 2%) and upper respiratory tract signs (1; 2%). On physical examination, fever was reported in 22 (49%) cats, pale mucous membranes in 14 (31%) cats, and jaundice in 14 (31%) cats. Dehydration was reported in 13 (29%) cats, and a fluid wave was palpable in 8 (18%) cats. A heart murmur was auscultated in 7 (16%) cats, and 1 cat was reported to have lymphadenopathy.

### 3.3. Laboratory and Diagnostic Imaging Results

The diagnosis of FIP was confirmed by RT-PCR in 31 (69%) cats and in 1 cat both by PCR and IHC. The other 13 (29%) cats had a presumptive diagnosis of FIP. All cats were either positive on saline agglutination (24; 53%) or Coombs test (13; 29%), or both (8; 18%). The titer for the Coombs test was available for 16 of 21 cats tested with a minimum titer of 1:16 to 1:512 for 15 cats and a 1:4 titer in 1 cat. The classification of cats by FIP diagnostic and anti-erythrocyte antibody methods is summarized in Table 1.

In most cats (40/45; 89%), IMHA was diagnosed at the time of diagnosing FIP; however, 5 cats were diagnosed with IMHA days later when the anemia was not resolving or was progressing despite antiviral therapy (8, 14, 16, 38, and 87 days after FIP diagnosis, respectively). Complete blood cell count was available for all 45 cats. The median hematocrit was 18% (IQR 13–20). However, since the blood samples had been assessed using different instruments with different reference intervals (RIs), the degree of anemia was normalized to the percentage below the lower limit of the RI; the median value percentage below the lower limit of the RI was 42% (IQR 30–56). Anemia was non-regenerative in 36 (80%) cats and regenerative in 5 cats; in 4 cats, the reticulocyte count was not available. Concurrent thrombocytopenia was present in 18 (40%) cats; 25 (56%) cats had a normal platelet count; and no platelet count was available in 2 cats. Neutrophilia was present in 12 cats; neutropenia was present in 7 cats; and 26 (58%) cats had normal neutrophil counts. Lymphocytosis was present in 4 cats and lymphopenia in 18 (40%) cats, and 23 (51%) cats had a normal lymphocyte count. Other abnormalities reported, based on microscopic blood smear examination, were hypochromasia in 9 cats, anisocytosis in 5 cats, polychromasia in 2 cats, and ghost cells in 1 cat. Two cats initially presented with hematocrit at the low end of normal RI but progressed to have anemia within several days of starting antiviral therapy.

Serum biochemistry profiles were available for all 45 cats. Albumin was low in 28 (62%) cats and normal in 17 cats. Hyperglobulinemia was reported in 29 (64%) cats, and 16 cats had normal globulin levels. Of the cats positive on saline agglutination, hyperglobulinemia was present in 14/24 (58%); hyperglobulinemia was present in 9/13 (69%) cats that tested positive on DAT alone and in 5/8 (63%) of cats that were positive on both. The median albumin to globulin ratio (A:G) was 0.3 (IQR 0.2–0.4). Hyperbilirubinemia was present in 34 (76%) cats (25/33 (76%) of the surviving cats and 9/12 (75%) of the cats that died), and other abnormalities reported were abnormal liver enzymes with increased ALT in 10 (22%) cats and increased ALP in 1 (2%) cat.

All cats were tested for FeLV antigen and FIV antibody using point-of-care (POC) tests (see Table 2), and 21 (47%) underwent hemoplasma PCR testing. One cat was FIV-positive (POC and Western blot), and this cat also tested positive for “*Candidatus* Mycoplasma haemominutum”. One cat each tested positive for FeLV antigen “*Candidatus* Mycoplasma turicensis” by PCR and *Anaplasma* spp. by PCR.

Thoracic radiographs were performed on 11 (24%) cats and showed pleural effusion (2 cats), lymphadenopathy (2), cardiomegaly (2), and pulmonary changes: broncho-interstitial (2), alveolar-interstitial (1), alveolar (1), and bronchial (1) patterns. There was increased pulmonary opacity (1) and pericardial effusion (1). Normal findings were described in 1 cat.

Abdominal ultrasound was performed on 37 (82%) cats. The majority of the cats (35/37; 95%) had more than one abnormality, with the most common findings being lymphadenopathy (31 cats; 84%), peritoneal effusion (26; 70%), renomegaly (10; 22%), splenomegaly (9; 20%), medullary rim sign (6; 13%), gall bladder thickening or edema (6; 13%), mottled spleen (5; 11%), heterogenous or hypoechoic liver (5; 11%), hyperechoic mesentery (5; 11%), thickened intestinal wall (4; 9%), hepatomegaly (3; 7%), splenic nodules (2; 4%), nodular pancreas (2; 4%), modular and edematous mesentery (1; 2%), enlarged pancreas (1; 2%), colonic mass (1; 2%), and pyelectasia (1; 2%).

### 3.4. Treatment and Follow-Up

All of the cats were treated with nucleoside analog antiviral medications: 39 (87%) with GS-441524 (GS), 3 cats with remdesivir and GS-441524, 2 with remdesivir, and 1 cat with molnupiravir. The majority of the cats (37/45; 82%) were treated with compounded antivirals. Eight (18%) cats were treated with unlicensed injectable GS-441524 sourced by the clients. This was prior to June 2024 when compounded medications were available in the USA. The dosage of remdesivir or GS-441524 was available for 38 (84%) cats, with a median dosage of 15 mg/kg/day (IQR 15–20). In the cat that was treated with molnupiravir, the dosage was 16 mg/kg every 12 h. The majority of the cats (35/45; 78%) were treated for 12 weeks, but in 10 (22%) cats the antiviral therapy was for 6 weeks. In most cats (39/45; 87%), prednisolone was started at presentation or within the first week of therapy; however, in 5 cats prednisolone was added later due to persistent anemia (8, 14, 16, 38, and 87 days, respectively). In 1 cat, the anemia resolved on GS therapy alone, and prednisolone was not started (this was the cat with lowest Coombs titer of 1:4). The prednisolone dosage was available for 36/44 (82%) of the cats, with a median dosage of 1.8 mg/kg/day (IQR1.1–2.0). A blood transfusion was given to 23 (51%) cats without any complications.

Other medications that were administered were antimicrobials (amoxicillin clavulanate (9 cats), doxycycline (4), clindamycin (3), pradofloxacin (1), marbofloxacin (1), enrofloxacin (1)) and secondary immunosuppressive medications (cyclosporine (5) and mycophenolate (2)). The cats that were started on secondary immunosuppressive treatments were started due to poor response on prednisolone alone. The dose of cyclosporine was 4/5–5 mg/kg BID, and the dose of mycophenolate was 8–10 mg/kg BID. Additional supportive treatments included antinausea/antiemetic medications, appetite stimulants, and clopidogrel. None of the 3 cats positive for “*Ca*. M. haemominutum”, “*Ca*. M. turicensis”, and *Anaplasma* spp. were treated with antimicrobials.

Median follow-up (from diagnosis) was 72 days (IQR 14–246). At the time of the latest follow-up, of the 45 cats, 33 (73%) cats had survived and 12 had died or were euthanized. Outcome was available for 31/33 cats that were alive (2 cats were lost to follow-up), and 1 cat was euthanized 320 days following diagnosis due to worsening of anemia and not responding to immunosuppressive therapy). Complete remission of FIP and IMHA occurred in 17/32 (53%) cats. In 3 (9.4%) cats, FIP remission was achieved, but IMHA relapsed, and in 1 of these IMHA relapsed twice. Two (6.2%) cats relapsed with FIP. In 1 (3.1%) cat, both FIP and IMHA relapsed. In 9 (28.1%) cats, the antiviral and glucocorticoid treatment was still ongoing at the time of publication. The thrombocytopenia resolved in all the cats that had concurrent thrombocytopenia at the time of diagnosis, and all of these cats were on prednisolone.

## 4. Discussion

This cohort of 45 cats represents the most comprehensive description to date of FIP-associated IMHA and the first published report documenting concurrent successful treatment of both conditions. The median age of cats in this study was 1.2 years, consistent with the finding that FIP usually occurs in younger cats, but also because cats with IMHA have been reported to be mainly younger to middle-aged cats [10,15,25,26]. The majority (76%) of cats affected were males, again consistent with previous reports of FIP as well as IMHA [10,15,25,26]. Almost half of the cats (47%) were purebred cats, with British Shorthair and Maine Coon cats being the most common. It has been reported previously that purebred cats are at higher risk for FIP, and these two breeds are amongst the most commonly reported breeds (they are also amongst the most popular cat breeds at present). However, no breed predisposition has been reported for IMHA [2,27]. Both FIP and IMHA occur mainly in young cats, and IMHA should be a considered particularly in young cats with FIP that are anemic, especially with moderate to severe anemia.

Associative IMHA was seen with both effusive and non-effusive FIP in this study, as well as with neurological and/or ocular involvement. It therefore appears that IMHA can occur with any form of FIP. The pathogenesis of IMHA in FIP is not known, but it is likely secondary to the severe systemic inflammation that is typical for FIP. On physical examination, the most notable signs reported in the cohort were fever, pale mucous membranes, and jaundice.

Clinical, hematologic, and biochemical parameters were similar to those previously reported in cats with FIP and also in cats with IMHA. The median hematocrit was 18%, slightly higher than the mean hematocrits reported in cats with IMHA of 12–15% [15,25,28]. Lymphocytosis was previously observed in 32% of cats with non-associative IMHA [15], while only 9% of cats in the current study had lymphocytosis at diagnosis. If there is a reduced prevalence of lymphocytosis when IMHA is associated with FIP, it could be because of the associative nature of the IMHA, occurring concurrently with FIP, which often results in lymphopenia [2,29]. A previous study reported anemia as a major clinical problem in 19/34 (56%) cats, and in 17 of these cats it reported the FIPV M1058L mutation and in another one both the M1058L and S1060A mutations, suggesting that anemia in FIP could possibly be related to some mutations in the FIP virus. Further studies should be performed analyzing whether cats with IMHA might have specific mutations in the virus leading to a higher incidence of immune-mediated anemia in cats with FIP.

Anemia was non-regenerative in 80% of the cats at the initial evaluation, which was unexpected for a diagnosis of immune-mediated destruction, but could also be explained by some of these cats having precursor-targeted immune-mediated anemia (PIMA), which is characterized by persistent non-regenerative anemia due to immune destruction of early precursors [30]. The lack of regeneration in associative IMHA could also be explained by inflammatory disease or concurrent bone marrow disorders, as approximately 50% of cats with FIP have non-specific reactive changes in their bone marrow at necropsy [31]. Alternatively, it is also possible that the cats had regenerative IMHA but were in an early stage of the disease; the bone marrow takes 3–5 days to respond to anemia [31]. Moreover, 40% of the cats had concurrent thrombocytopenia, which was previously reported in cats with IMHA and PIMA [15,25,32]. In addition to immune-mediated destruction, thrombocytopenia may be caused by sequestration of platelets within the enlarged spleen of these cats or consumption due to hypercoagulability progressing to disseminated intravascular coagulation (DIC). However, DIC was not suspected in any of the cats in this study based on their clinical examination and diagnostic investigations.

On serum biochemistry, hyperbilirubinemia and hyperglobulinemia were frequently seen in the cats in this study. Hyperbilirubinemia was present in 76% of the cats and has been previously reported in 68% of cats with IMHA and in around 25% of cats with FIP [15,25,33]. This is likely pre-hepatic due to increased breakdown of hemoglobin and/or hepatic due to the severe inflammation in the liver of these cats (increased ALT activity was reported in 22% of the cats in this study, which could reflect hepatic involvement in FIP and/or possible hepatic hypoxemia secondary to anemia). Increased ALT activity has been previously reported both in cats with FIP and IMHA as well [25,29]. Both hyperbilirubinemia and increased ALT can be due to FIP or IMHA, but the increased incidence in this study is likely due to the combination of IMHA and FIP. Hyperbilirubinemia has been reported as a poor prognostic indicator in cats with IMHA and FIP [15,34]; however, in this study both live and dead cats had hyperbilirubinemia (76% of live cats and 75% of the cats that died), suggesting that hyperbilirubinemia in FIP cats with associative IMHA might not be a poor prognostic indicator. Hyperglobulinemia was reported in 64% of the cats in this study and has been reported previously in cats with FIP and also with IMHA [15,25]. In both FIP and IMHA, this could be due to chronic stimulation of the immune system.

Additional infectious agent-testing reported co-infections in 4 cats, and it is possible that these infections may have contributed to their anemia and/or triggered associative IMHA as well as FIP; however, according to the ACVIM consensus statement on the diagnosis of IMHA in dogs and cats, there is very limited evidence that “*Ca*. M. haemominutum” or “*Ca*. M. turicensis” can cause associative IMHA in cats [24]. Additional infections were unlikely to be the primary cause of the IMHA in the cats in this study, as they were only found in a small number of the cats and not deemed to be the cause of the IMHA in these cats; however, not all cats were tested for hemoplasmas in this study. Given the other abnormalities in the cats not tested for hemoplasmas, as well as the response to antiviral therapy and glucocorticoids, it is very likely that the IMHA was associative to FIP in these cats.

Abdominal ultrasound showed multiple abnormalities in 95% of the cats in which it was performed, with the most common findings being lymphadenopathy, peritoneal effusion, renomegaly, and splenomegaly. Other studies have reported splenomegaly with non-associative IMHA, in 60% of cats in one study [28] and 42% of cats (with homogeneous parenchyma reported) in another study [25]. Diffuse splenomegaly can be caused by inflammatory or infectious processes (e.g., FIP, bartonellosis, and toxoplasmosis), infiltration of abnormal cells (e.g., neoplasia), increased extramedullary hematopoiesis, congestion, or hyperplastic splenomegaly (e.g., related to hemolysis) [35]. In this study we only noted splenomegaly in 24% of the cats, which is interesting because it can be caused by both IMHA and FIP, and this could further support the hypothesis that PIMA was more common in this study cohort, given the non-regenerative anemia, and that PIMA may not stimulate as robust a splenic response.

At the time of publication, 73% of the 45 cats with FIP and associated IMHA had survived, which is only slightly lower than for the cats with FIP in which IMHA was not described [2,8,29]. A mortality rate of 27% is similar to the rates of 24% in other studies of cats with IMHA [25]. This could suggest that IMHA, not FIP, is the outcome-defining factor in these cats with both diseases.

This study has a number of limitations, the main one being that it is a retrospective multicentric study with some missing data or tests not being performed (e.g., follow-up information and diagnostic investigation results). There was also variation in the diagnostic imaging and infectious disease testing performed due to its multicentric nature. Therefore, blood samples were submitted to different laboratories which used different methodologies and RIs, making direct comparisons challenging. This is why the study reports the percentage of cats below the RI for the hematocrit values, as well as describing the median and IQR to give an indication of the dispersion of the data. However, the multicenter design was necessary to obtain as many case reports as possible, and it is a rare example of a worldwide scientific collaboration on a very severe disease to accumulate knowledge and help as many cats as possible.

Another limitation is that not all cats had their diagnosis of FIP confirmed by PCR or IHC; however, a presumptive diagnosis of FIP can be made based on signalment, clinical signs, and diagnostic investigations, as well as response to antiviral therapy.

There are also concerns about the utility of Coombs testing (DAT) and/or agglutination in saline in the diagnosis of IMHA. Single positive test results of these assays in people or cats may be related to factors other than development of antibodies against erythrocytes, including viral infections like human immunodeficiency virus and non-specific erythrocyte binding secondary to multiple causes of hyperglobulinemia [36,37,38,39]. However, as cats with non-associative IMHA can also present with hyperglobulinemia, this is a general limitation for all cats with IMHA, and this study’s criteria follow the ACVIM consensus on diagnosis of IMHA [15,24]. That said, Coombs testing for the confirmation of IMHA is controversial in both dogs and cats, mainly because it does not distinguish between non-associative and associative IMHA. Positive testing does not verify whether there are antiautologous antigens or antibodies against red cell-attached xenoantigens, such as drugs or components of infectious agents or immune-complexed antibodies bound non-specifically to the surface of red blood cells. Another limitation of Coombs testing in a multicentric study is that different reagents may have been used for different cases. However, false positives are rare; a study looking at Coombs testing for the validation of IMHA found that no healthy or sick non-anemic cats had a positive Coombs test, and only 1 of the 56 cats (1.8%) with blood loss anemia or non-regenerative anemia due to renal failure, retrovirus infection, or anemia of inflammatory disease had a positive Coombs test [25]. Additionally, all of the 7 cats in the same study that were diagnosed with hemolysis not related to immunological processes, such as hypophosphatemia or Heinz body anemia, were Coombs test-negative; this included 2 cats with FIP [25]. However, it is not clear if the other anemic or healthy cats had hyperglobulinemia in this study. In another study looking at Coombs testing in cats, only 3% of non-anemic cats were Coombs positive at 37 °C, and these cats both had high titered reactions (at both 4 °C and 37 °C) with polyvalent antiserum and anti-IgG, and both were diagnosed with pancreatitis [26]. On the other hand, only including cats that were positive on saline agglutination or Coombs testing could have led to an exclusion of some cats, e.g., those with PIMA, which can have immune-mediated destruction but test negative on these tests, as has been reported previously [30].

There were a number of other limitations. None of the cats had bone marrow analysis performed, which could have further helped in evaluating the non-regenerative anemia in these cases. This was likely due to the severity of the cats’ clinical signs precluding more invasive procedures and/or a reluctance to perform this procedure on small patients; because of this, bone marrow diseases could not be ruled out. However, IMHA was mostly diagnosed due to the presence of persistent agglutination and/or by the direct antiglobulin Coombs test, according to the ACVIM consensus statement on the diagnosis of IMHA in dogs and cats [24]. Pyruvate kinase deficiency and increased osmotic fragility of erythrocytes as hereditary causes for hemolysis were very unlikely because of the positive Coombs test results obtained in many cats and breed predisposition, as there were no Abyssinian or Somali cats in this study. Additional causes of hemolysis (e.g., hypophosphatemia and Heinz body hemolytic anemia) were excluded by serum biochemistry and evaluation of blood smears. It was not possible to determine what effect the dosages and duration of treatments had on prognosis, as different treatments and dosage regimens were involved. Lastly, some cats were lost to follow-up or still in treatment at the time of publication, further limiting survival time data.

## 5. Conclusions

In conclusion, this study is the first to describe a large cohort of cats with both FIP and associative IMHA, which appeared to be more common than previously reported in cats with FIP. It is also important to note that the majority of the cats had non-regenerative anemia, and an immune-mediated cause of anemia in cats with FIP should be considered also with non-regenerative anemia. The findings of this study should ideally be confirmed in a prospective study, which would permit assessment of a complete workup for concurrent infections, the efficacy of different treatment protocols, and tapering dosage regimens. Although FIP has been an uncommon cause of associative IMHA, as more cats with FIP are successfully treated with antiviral therapy, it is important to consider IMHA as a possible cause of anemia in cats with FIP.

## Figures and Tables

**Table 1:** Distribution of FIP and IMHA classification results.

		IMHA Testing Positive		
FIP Diagnosis		Saline Agglutination	Coombs	Both
Confirmed	32	16	10	6
Presumed	13	8	3	2
Total	45	24	13	8

**Table 2:** Distribution of concurrent infectious agent testing results.

		Infectious Disease Testing		
FIP Diagnosis		FIV/FeLV Negative	Hemoplasma Performed	Hemoplasma Negative
Confirmed	32	30	12	11
Presumed	13	13	9	8
Total	45	43	21	19

## Data Availability

The data from this study are not currently available as they are still being studied. Once this work has been completed, the data will become available.

## Abbreviations

The following abbreviations are used in this manuscript:

IMHA	Immune-mediated hemolytic anemia
RT-PCR	Reverse transcription polymerase chain reaction
DAT	Direct antiglobulin test
PIMA	Precursor-targeted immune-mediated anemia
